# Monitoring sertraline and clozapine levels

**DOI:** 10.1192/j.eurpsy.2022.1852

**Published:** 2022-09-01

**Authors:** A. Rodriguez Campos, L. Rodriguez Andrés, G. Medina Ojeda, I. Santos Carrasco, J. Gonçalves Cerejeira, A. Gonzaga

**Affiliations:** 1 Hospital Clinico Universitario de Valladolid, Departamento De Psiquiatría, Valladolid, Spain; 2 Clinical Hospital of Valladolid, Psychiatry, Valladolid, Spain; 3 Hospital Clínico Universitario de Valladolid, Psychiatry, Valladolid, Spain; 4 HOSPITAL CLINICO UNIVERSITARIO DE VALLADOLID, Psiquiatria, VALLADOLID, Spain

**Keywords:** sertraline clozapine side effects

## Abstract

**Introduction:**

One of the most frequent side effects seen when prescribing sertraline and clozapine together is the appearance of a seizure crisis. This event is usually related to an increase of plasmatic concentration due to interactions of these drugs with blood components.

**Objectives:**

To investigate the effects of clozapine when combined with other drugs, especially its effects increasing plasmatic concentration.

**Methods:**

A patient was treated sith 300 mg/day of clozapine followed by a treatment with sertraline 50 mg/day, which increases plasmatic concentration. The combination of these treatments produced seizures. Other works published about interactions are reviewed.

**Results:**

It is important to monitor clozapine dosages to avoid increasing plasmatic concentration, especially if other drugs that have this effect are also administered.

**Conclusions:**

It is important to monitor clozapine dosages to avoid increasing plasmatic concentration, especially if other drugs that have this effect are also administered.

**Disclosure:**

No significant relationships.

